# Fracture resistance and the mode of failure produced in metal-free crowns cemented onto zirconia abutments in dental implants

**DOI:** 10.1371/journal.pone.0220551

**Published:** 2019-08-08

**Authors:** Rubén Agustín-Panadero, Blanca Serra-Pastor, Ana Roig-Vanaclocha, Antonio Fons-Font, María Fernanda Solá-Ruiz

**Affiliations:** Department of Dental Medicine, Faculty of Medicine and Dentistry, Valencia University, Valencia, Spain; Meridional Faculty IMED, BRAZIL

## Abstract

The purpose of the investigation was to analyze fracture resistance and mode of failure of zirconium oxide (zirconia) abutments placed on dental implants bearing crowns of different esthetic materials: zirconia, lithium disilicate (LDS), and nano-ceramic resin, for replacing single teeth in the anterior sector. Eighty implant-abutment-crown units were divided into four groups: Group T-MC (control): 20 metal-ceramic crowns cemented onto titanium abutments; Group Z-Z: 20 zirconia crowns on zirconia abutments; Group Z-LD: 20 lithium disilicate crowns on zirconia abutments; and Group Z-NCR: 20 nano-ceramic resin crowns on zirconia abutments. Specimens underwent a fatiguing process (dynamic loading and thermocycling), followed by static loading to evaluate mechanical fracture resistance, and the mode of failure produced. Mean fracture resistance values were: Control Group T-MC, 575.85±120.01 N; Group Z-Z 459.64±66.52 N; Group Z-LD, 531.77±34.10 N; and Group Z-NCR, 587.05±59.27 N. In Group T-MC, fracture occurred in the prosthetic fixing screw in 100% of specimens. In Group Z-Z, 80% of fractures occurred in the fixing screw, 15% in the abutment, and 5% in the abutment and crown. In Group Z-LD, 60% of fractures were produced in the fixing screw and 40% in the abutment. In Group Z-NCR, 70% of fractures were produced in the fixing screw and 30% in the abutment. All the abutments and crowns analyzed have the potential to withstand the physiological occlusal forces to which they would be subject in the anterior region. Lithium disilicate and nano-ceramic resin crowns cemented onto zirconia abutments are a good restoration alternative for single implants in the anterior sector.

## Introduction

A single missing tooth in the anterior sector is a complex challenge to the dentist, due to the need for restorations that are both esthetic and functional. One of the classic treatment options of choice is an implant-supported restoration with titanium abutment and metal-ceramic crown, which enjoys a high success rate of around 95% [1–3].

Today, zirconia is considered a suitable material for prosthetic rehabilitation of anterior teeth due to its high biocompatibility, its esthetics (white color), and its mechanical properties, as it can withstand masticatory forces of over 150N [4–9].

In response to the esthetic requirements of the anterior sector, ceramic materials have come into use for fabricating implant abutments. In 1993, Prestipino and Ingber designed and used an aluminum oxide abutment for the first time in a single-implant rehabilitation. But aluminum oxide has been found to present lower mechanical resistance to the occlusal forces exerted in the anterior sector in both *in vitro* and *in vivo* trials, and for this reason, in recent years, zirconium oxide has been proposed as a material for fabricating this type of abutment [10]. Zirconia abutments appear to offer adequate biocompatibility as well as optical properties that make it possible to obtain a peri-implant mucosa color that is similar to the gum around a natural tooth. Nevertheless, some doubts remain as to the material’s fracture resistance and its mechanical behavior in the intraoral environment. *In vitro* trials of zirconia’s fracture resistance have obtained widely varying results, with values ranging from 131 to 2517 N. Sailer [11] conducted a systematic review of clinical studies of zirconia abutment behavior, finding that the main problem is their mechanical response to traction forces. But more recently, new laboratory studies have been published evaluating fracture resistance of anterior restorations with zirconia abutments, obtaining values above the occlusal forces exerted in the anterior sector (300 N) [1,6,12]. Therefore, the aim of this *in vitro* study was to analyze the fracture resistance and mode of failure produced (after fatiguing) of zirconia abutments bearing crowns fabricated from different esthetic materials: zirconia, lithium disilicate (LDS), and nano-ceramic resin, compared with the classic materials titanium abutments bearing metal-ceramic crowns used to replace a single missing anterior tooth.

## Materials and methods

### Materials

Eighty tapered implants were used (Khono, Sweden&Martina¡, Padua, Italy), 11.5 mm long with a diameter of 4.25 mm, with internal hexagonal prosthetic connections 2 mm in depth. The specimens were divided into four groups according to the crown and abutment materials:

- Group T-MC (control group) (N = 20 specimens) consisting of titanium prosthetic implant abutment screwed to the implant and a metal-ceramic crown cemented onto the abutment. The metal-ceramic crowns had Cr-Co cores fabricated using CAD-CAM technology (3Shape CAD Design Software, Copenhagen, Denmark) with a press veneering porcelain (IPS Ceramic, InLine Pom, Ivoclar Vivadent, Schann, Liechtenstein).
- Group Z-Z (N = 20 specimens): a zirconia abutment screwed to the implant, and a crown with a zirconia core and a press veneering porcelain (IPS Ceramic, InLine Pom, Ivoclar Vivadent) cemented onto the abutment.
- Group Z-LD (N = 20 specimens) consisting of a zirconia abutment screwed to the implant and a monolithic lithium disilicate ceramic crown screwed onto the abutment. The lithium disilicate crowns were designed and fabricated using CAD/CAM technology from pre-sintered blocks (IPS e.max CAD, Ivoclar Vivadent).
- Group Z-NCR (N = 20 specimens) consisting of a zirconia abutment screwed to the implant and a nano-ceramic resin crown cemented onto the abutment. The nano-ceramic resin crowns were designed and fabricated using CAD/CAM technology (Lava Ultimate, 3M ESPE, Diegem, Belgium).

**Fig 1:**
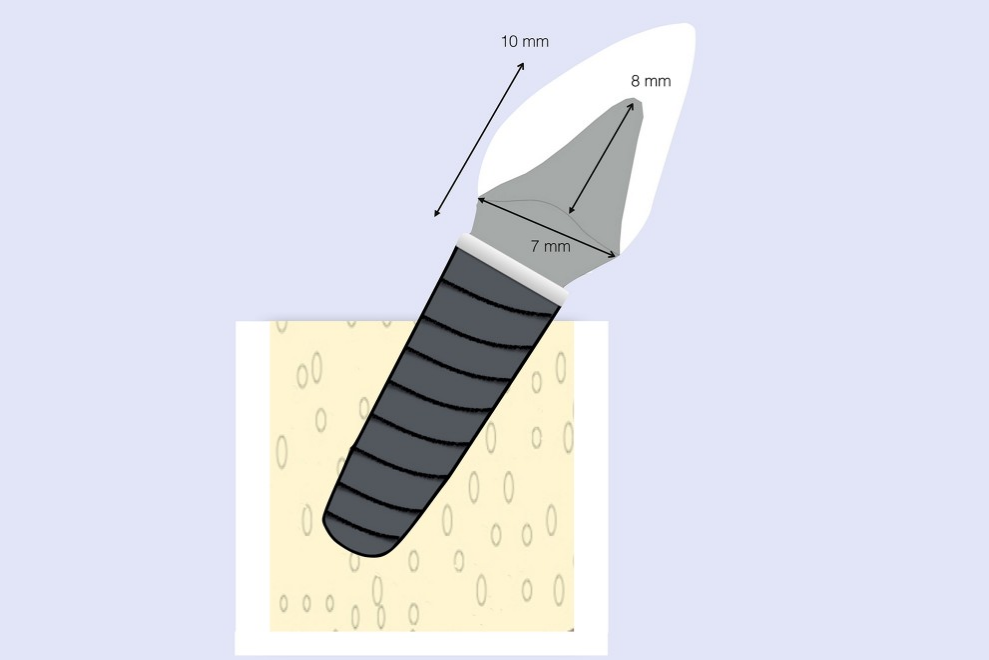
Design of the abutments with standardized parameters.

All the abutments analyzed in the study were designed using CAD/CAM Echo2 software (Sweden&Martina) with standardized parameters. The total height of the abutment (from the implant’s prosthetic platform to the most incisal part) was 10 mm. The abutment’s clinical height (from the finish line to the most incisal part) was 8 mm. The abutment’s finish line diameter was 7mm (Fig 1). The angle of the abutment axial convergence was 6°. The finish line was chamfered and of 1 mm width. The titanium abutments (one-piece, abutment and connection) were milled from blocks of titanium (Bio-Titanium, Sweden&Martina). Zirconium dioxide abutments were made up of two parts, a milled pre-sintered zirconia core and a T-Connect titanium connection (Sweden&Martina) which was cemented to the cervical area of the zirconia abutment using composite resin cement (RelyX Unicem 2, 3M ESPE).

In vitro fracture resistance evaluation followed the methods established in UNE-EN ISO 14801:2007. The implants were set in epoxy resin (Exakto-Form; Bredent) with an elastic modulus of over 3 GPa, inside nylon tubes. To simulate peri-implant bone loss, 3 mm of the implant was left exposed from the implant collar in root-crown direction; the implants were set at an angle of 30° to the direction of loading [13].

When all the implants had been set in tubes, the abutments were screwed onto the implants using a type IV titanium screw applying a torque of 30 N/cm with a prosthetic screwdriver equipped with a torque control device ISD900 (NSK, Tokyo, Japan). Access chimneys to the abutment screws were sealed with polytetrafluoroethylene (PTFE). Before cementation, the zirconia abutment, and the internal surfaces of the zirconia, lithium disilicate, and nano-ceramic resin crowns were prepared in various ways. All zirconia abutments were sand-blasted with 30μm silicon oxide particles (SiO2), at 2 bars pressure from a distance of 10 mm, using a Cojet Sand clinical sand-blaster (3M ESPE). These were then primed with silane (RelyX Ceramic Primer, 3M ESPE). The zirconia crowns were also sand-blasted using the same technique as the abutments. The lithium disilicate crowns were etched with 9.6% hydrofluoric acid (Ultradent porcelain Etch) for 20 seconds and then, after washing and drying, brushed with silane (RelyX Ceramic Primer, 3M ESPE). The nano-ceramic crowns were sand-blasted with 50 μm aluminum oxide particles at 2 bars pressure from a distance of 10 mm (Cojet Sand, 3M ESPE) applying silane afterwards. The titanium abutments did not receive any surface treatment before cementation. Metal-ceramic crowns were sand-blasted with aluminum oxide particles (Cojet Sand, 3M ESPE).

**Fig 2:**
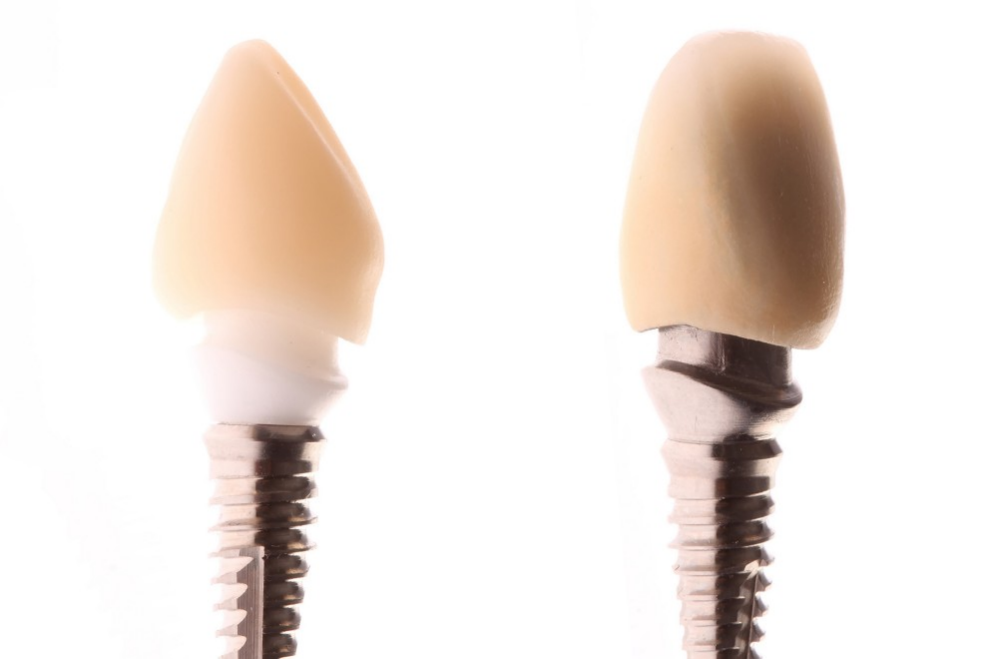
Metal-free restoration in zirconia abutment and metal-ceramic restoration in titanium abutment.

The crowns were cemented (Fig 2) onto their corresponding abutments with dual-cure resin cement (RelyX Unicem 2; 3M ESPE). The cement was applied to the axial walls inside each Crown and the crown placed on the abutment. When in place on the abutments and before cement curing, the crowns were subjected to a force of 1 kg to seat the crown correctly on the abutment and for optimal distribution of the cement. Halogen light was applied for 60 seconds to initiate the curing process. After 120 seconds, excess cement was removed from the abutment-crown interface. The 1 Kg load was maintained for a total of 5 minutes [14].

### Method

**Fig 3:**
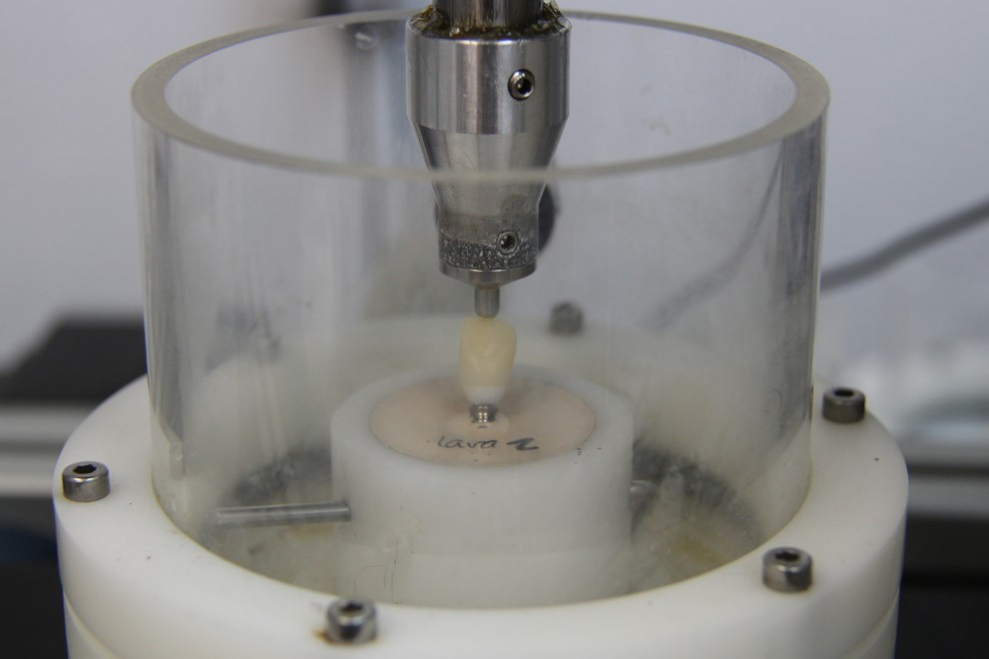
Area of load application in the fatigue test.

All specimens underwent an aging process consisting of dynamic loading and thermocycling. Dynamic loading was performed using a chewing simulator (CS-4.2 economy line; SD Mechatronik GMBH, Feldkirchen-Westerham, Germany) and consisted of 240,000 masticatory cycles applying a vertical load of 8 kg with a vertical movement of 2.5 mm and horizontal movement of 2 mm below the crowns’ incisal edges. At the same time as dynamic loading, the specimens were subjected thermocycling (Thermocycling TC-3; SD Mechatronik GMBH), 1,548 thermal cycles per specimen, with changes in temperature from 5° to 55° every 30 seconds (Fig 3).

After fatiguing and thermocycling, all specimens (crown-abutment) were tested by static load compression to evaluate their mechanical strength. A Shimadzu AG-100KN universal test machine (Shimadzu corporation) was used applying a cell load of 5000N; the cross head speed was 0.5 mm/min and the load was applied until fracture of the crown-abutment structure took place.

When compression testing to the point of fracture had been performed, each specimen was examined under an optical microscope (Leica Microsistemas S.L.U.; Barcelona, Spain) to analyze the type of fracture and its location, classifying the fractures as follows: crown fracture without abutment fracture; abutment fracture without crown fracture; fracture of both crown and abutment; fixing screw fracture.

All the data obtained were processed with TRAPEZIUM-X software (single serial 942356CA, Shimadzu corporation). Descriptive statistics were calculated for the force variable: mean, standard deviation, minimum, maximum, and median. Confidence intervals of 95% were computed for mean values. A one-way ANOVA general linear model (GLM) was developed to determine if the mean level of fracture resistance could be considered homogeneous or not between groups. The significance level used in analysis was 5% (α = 0.05). Multiple comparisons were made using the Bonferroni test.

## Results

### Dynamic loading results

During the application of dynamic load cycles and thermocycling, none of the specimens suffered fractures, delamination, or screw loosening. In this way, all specimens were available for fracture resistance evaluation (Table 1).

**Table 1:** Load resistance and type of mechanical failure.

**N**				***GROUP***				
	*T-MC*		*Z-Z*		*Z-LD*		*Z-NCR*	
	*LOAD (N)*	*FRACTURE*	*LOAD (N)*	*FRACTURE*	*LOAD (N)*	*FRACTURE*	*LOAD (N)*	*FRACTURE*
1	557,153	Screw	521,247	Screw	564,098	Screw	556,04	Screw
2	562,493	Screw	414,356	Screw	551,494	Screw	512,06	Abutment without crown
3	592,105	Screw	447,877	Screw	581,328	Screw	613,642	Screw
4	590,928	Screw	474,151	Screw	432,158	Abutment without crown	627,422	Abutment without crown
5	602,945	Screw	546,694	Abutment with crown	529,941	Screw	799,942	Screw
6	758,028	Screw	421,937	Screw	538,476	Screw	585,191	Screw
7	534,63	Screw	495,497	Screw	532,039	Screw	579,929	Screw
8	493,272	Screw	475,264	Screw	546,042	Screw	621,478	Screw
9	616,916	Screw	394,932	Abutment without crown	528,733	Screw	573,858	Screw
10	628,805	Screw	484,419	Screw	533,756	Screw	583,14	Abutment without crown
11	857,306	Screw	419,919	Screw	593,408	Screw	556,771	Abutment without crown
12	493,336	Screw	426,07	Screw	511,583	Screw	530,593	Screw
13	457,764	Screw	527,159	Screw	521,771	Abutment without crown	626,898	Screw
14	790,024	Screw	355,609	Abutment without crown	549,905	Abutment without crown	568,692	Screw
15	497,119	Screw	439,994	Screw	546,662	Screw	534,646	Abutment without crown
16	581,106	Screw	389,226	Screw	498,788	Abutment without crown	576,242	Screw
17	597,127	Screw	589,943	Screw	488,822	Abutment without crown	582,711	Screw
18	425,959	Screw	431,267	Screw	530,259	Abutment without crown	596,603	Abutment without crown
19	523,091	Screw	365,671	Abutment without crown	518,068	Abutment without crown	570,52	Screw
20	356,897	Screw	571,489	Screw	537,984	Abutment without crown	544,691	Screw

**Fig 4:**
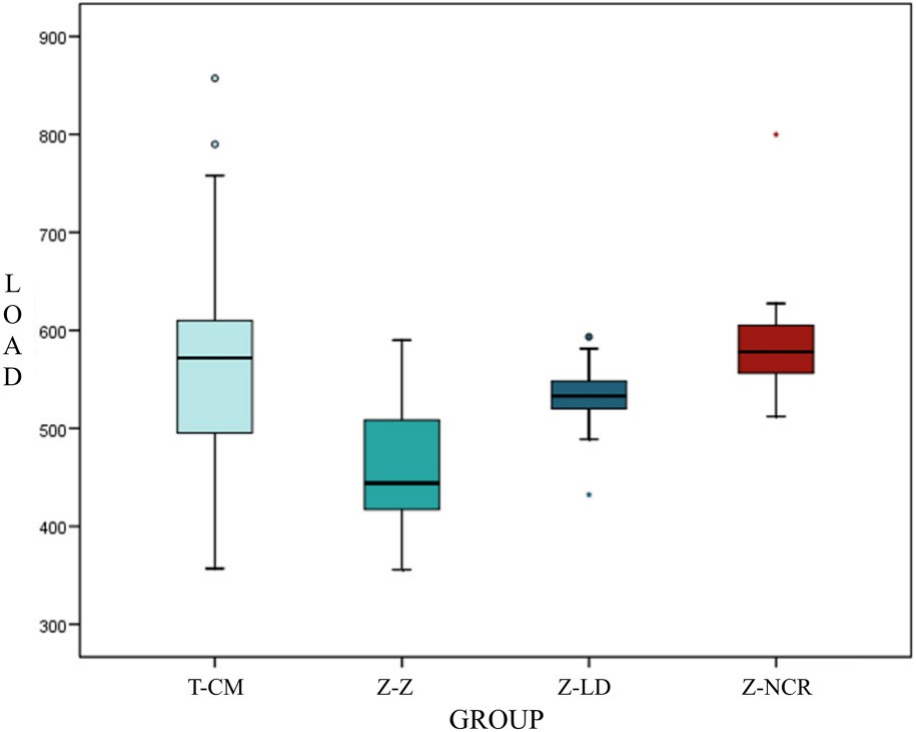
Box plot representing fracture resistance (N) of each group.

Fracture resistance evaluation obtained the following mean values: For group T-MC, mean resistance was 575.85±120.01 N; Group Z-Z 459.64±66.52 N; group Z-LD 531.77±34.10 N; and for Group Z-NCR mean fracture resistance was 587.05±59.27 N (Fig 4).

One-way parametric ANOVA tests were used to determine homogeneity and statistic significant differences in resistance between the groups (Table 2). The Bonferroni test confirmed that Group Z-Z obtained significantly lower fracture resistance values in comparison with the other three groups: Z-Z vs. T-CM (p-value <0.001); Z-Z vs. Z-LD (p-value p = 0.024); Z-Z vs. Z-NCR (p-value <0.001). In other comparisons, no statistically significant differences were identified: T-CM vs. Z-LD (p = 0.0437); T-CM vs Z-NCR (p-value = 1); Z-LD vs Z-NCR (p-value = 0.152).

**Table 2:** One-way parametric ANOVA tests to determine homogeneity and statistic significant differences in resistance between the groups.

	Sum of squares	Degrees of freedom	Quadratic mean	F-statistic	Significance
Intergroup	200346’487	3	66782’162	11’365	0’000
Intragroup	446582’503	76	5876’086		
Total	646928’990	79			

### Fracture analysis results

**Fig 5:**
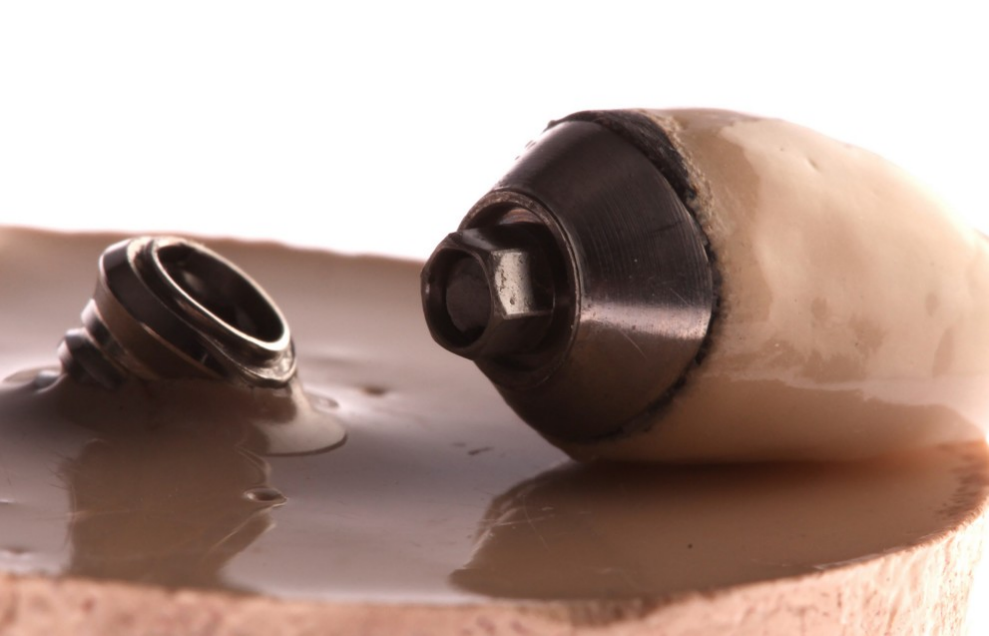
Fracture in the fixing screw of the T-CM group.

When the type of fracture and fracture location were analyzed, the following overall distribution was observed: 77.5% of all fractures were in the fixing screw between implant and abutment (62 fractures), 21.3% were located in the abutment but this only occurred among specimens with zirconia abutments (17 fractures), and 1.3% were located simultaneously in the crown and the abutment (1 fracture). In the T-CM control group, no fractures occurred in the abutment; all specimens (100%) suffered fractures in the fixing screw (Fig 5).

**Fig 6:**
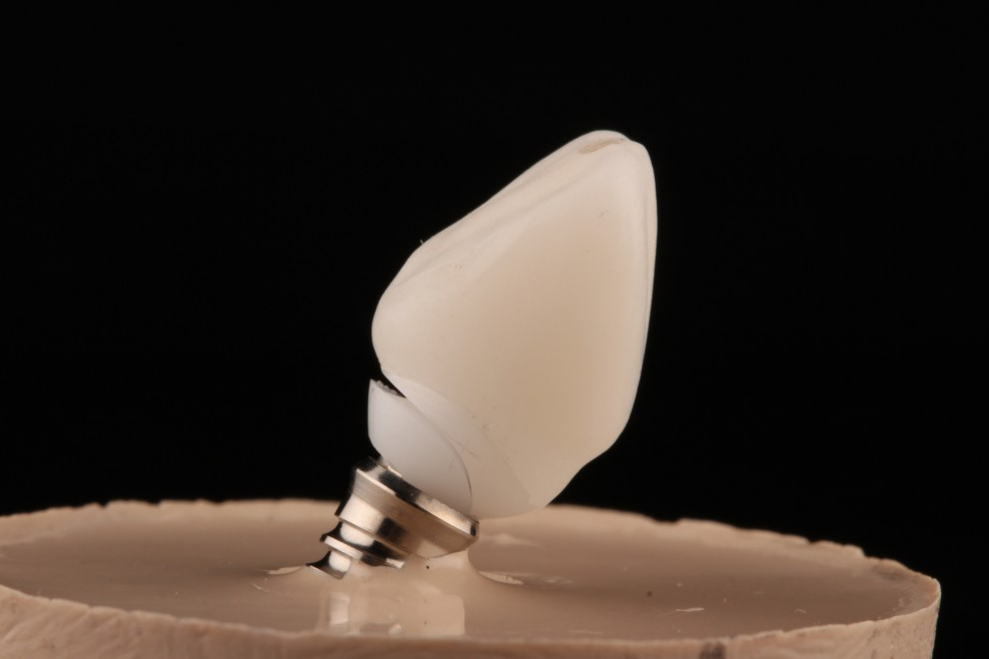
Fracture in the abutment of the Z-LD group.

In the Z-Z Group, most fractures were produced in the screw (80%), although in three specimens out of 20 (15%) fracture was located in the abutment; and in one case (5%), both the abutment and crown fractured. In the Z-LD Group most fractures were located in the fixing screw (60%) and the rest in the abutment (40%) (Fig 6).

**Fig 7:**
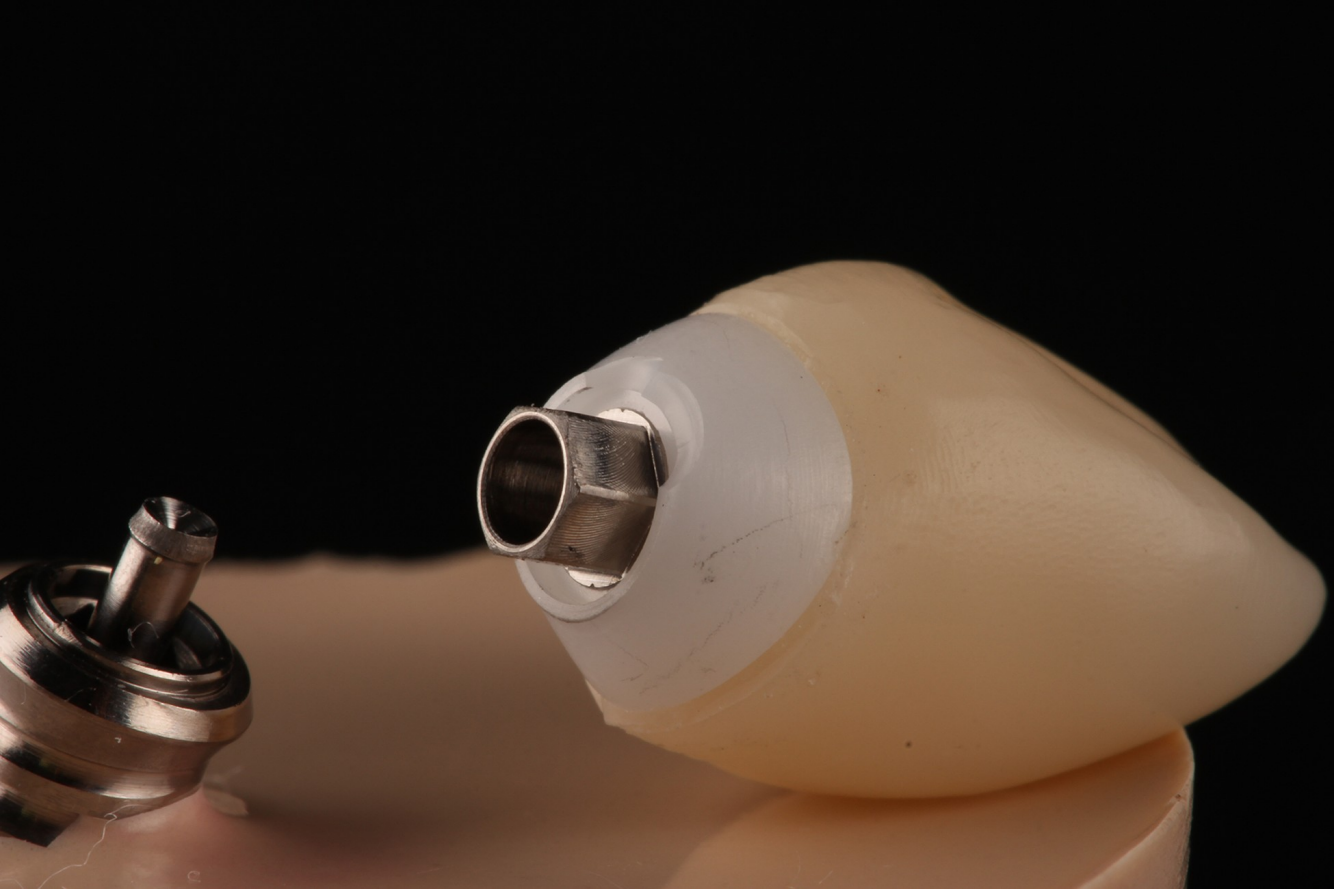
Fracture in the fixing screw of the Z-NCR group.

In the Z-NCR Group, most specimens suffered fracture in the screw (70%) (Fig 7), and the rest in the abutment (30%). The Kruskall-Wallis test confirmed statistically significant differences in the type of fracture between groups (Chi^2^ = 10.614).

## Discussion

The present study analyses the fracture resistance of zirconia abutments on implants, as well as the type of fracture produced. The zirconium dioxide abutments were restored with crowns fabricated from different materials, comparing these with the classic combination of titanium abutment bearing a metal-ceramic crown (Control Group), intended to replace a single missing tooth in the anterior sector. The study design followed norms established in UNE-EN ISO 14801:2007 for trials of endosseous dental implants subjected to dynamic fatiguing. The procedures followed have been used in many other published studies [6,13,14].

To reproduce the conditions that restorations are subject to in the oral medium (mechanical masticatory stress, temperature changes) specimens underwent an artificial ageing process consisting of dynamic loading and thermocycling. The literature includes various *in vitro* studies [6,15–17] that have followed this type of procedure for evaluating the fracture resistance of ceramic materials such as zirconia, as they can suffer decreases in strength due to low temperature degradation (LTD). This involves a transition in the zirconia phase (from tetragonal to monoclinic phase) resulting from repeated thermal stress to the restorations [18].

Analyzing the fracture resistance data obtained in the study, the control group specimens with titanium abutment and metal-ceramic crown (T-MC Group) obtained mean values of 575.85±120.01N, which are similar to values obtained by Protopapadaki [19] (525.89±143.547 N). But a study by Foong [20] registered much lower values (270±56.7 N), while a study by Mühlemann [16] obtained extremely high values (1042±86.8N). The zirconia abutment with zirconia crown group (Z-Z Group) (459.63±66.52 N) obtained very similar fracture resistance values to a study by Att^7^ (475±252N), but Gehrke [6] and Joo [8] obtained much lower values, 291.4±27.8N and 292.74±37.15N respectively. Kim [9] obtained very high values (729.2±35.9N). The lower fracture resistance found in the present study’s Z-Z group could be due to the rigidity of the abutment-crown union that would affect force distribution in the whole restoration-implant complex. The presence of a material like zirconia with its low deformation capacity increases the transmission of forces to implant’s prosthetic connection. In the zirconia abutment and lithium disilicate crown group (Z-LD Group), mean fracture resistance was 531.77N, a similar result to that obtained by Mühlemann [16] (531,77 N), while Elsayed [1] obtained a very high mean value of 944N. An *in vitro* trial by Martínez-Rus et al. compared the fracture resistance of zirconia abutments supporting monolithic lithium disilicate crowns, lithium disilcate crowns with a manually-applied esthetic covering, and zirconia crowns, all reproducing the anatomy of the upper central incisors. The results showed that the zirconia abutments supporting both types of disilicate crown obtained slightly higher results (363–392 N) than samples restored with zirconia crowns (340 N) [21].

Lastly, the zirconia abutment with nano-ceramic resin crown group (Z-NCR Group), obtained a fracture resistance value of 587.05±59.27 N, which cannot be compared with any other research, as no other literature has dealt with this variable for zirconia abutments and crowns of this hybrid material. Authors such as Ferrario et al. [22], have found that the occlusal forces exerted on a single tooth in a healthy adult male are of 140 and 150 N for central and lateral incisors respectively. But Gibbs et al. [23] claim that the occlusal load is of 263 N during normal mastication. Another study by Waltimo & Könönen [24] state that average maximum force is 263 N for men and 243 N for women, given that the maximum physiological bite force for incisors may reach 290 N depending on facial morphology and age [25]. Waltimo [26] reports that maximum bite force in the anterior sector could be as high as 569 N in the presence of parafunction.

Based on these results, we can recommend the use of zirconia abutments in the anterior sector, especially in cases where, due to lack of bone, the implant has been positioned inclined to vestibular. In these case it is important to choose the material not only by their mechanical properties, but aesthetic ones. Thin thickness of the gingiva at the cervical level, in these situations, can make the material positioned under the gum visible. Zirconia is a white material, so it masks better than titanium under the gum, and also provides high strength values for anterior sector. The use of this type of abutment in the posterior sector would be extremely risky given the mechanical requirements in this area.

The types of fracture registered were as follows: in the titanium abutment and metal-ceramic Crown group (T-MC Control Group), fractures occurred in the prosthetic fixing screw in all samples, a finding that coincides with Sghaireen [27], who obtained very similar results. In the zirconia abutment with zirconia crown (Z-Z Group), most of the specimens suffered fracture in the screw (80%), although in 15% of the specimens, fracture occurred in the abutment and in one specimen the fracture was located in the abutment-crown complex, findings that concur with Att [7], although the latter study did not use the same method. In the zirconia abutment and lithium disilicate group (Z-LD Group), 60% of the specimens underwent fracture in the fixing screw and in the remaining 40% fractures occurred in the abutment. These results are similar to those published by Kim [28], who observed that most fractures were located in the screw or the abutment. As for fracture type in the zirconia abutment with nano-ceramic resin crown group (Z-NCR Group), in 70% of the specimens, fractures were located in the screw and the remaining 30% in the abutment. These findings cannot be compared with any other published literature as none have evaluated crowns of this material cemented onto zirconia abutments.

The majority of the studied samples fractured at the level of the abutment-implant union screw, so the results obtained from the fracture analysis can not be argued with the parameters studied in this article (behavior of the restoration materials). The fracture of the screw depends on other variables such as screw material, type and height of implant connection, screw insertion torque and others.

The main limitation of our study was its own nature, be an in vitro study. Although attempts have been made to reproduce the conditions of the oral environment, the results can not be extrapolated to 100% at the clinical level. We think that this research should be continued with a higher level of evidence by conducting a clinical trial.

## Conclusions

Within the limitations of this study, it may be concluded that: All the abutments and crowns fabricated from the materials evaluated in the present *in vitro* study have the potential to withstand the physiological occlusal forces that occur in the anterior region. The group presenting the lowest fracture resistance was the group of zirconia abutments bearing zirconia crowns (Group Z-Z) with statistically significant differences in comparison with the other groups. Lithium disilicate crowns and nano-ceramic resin crowns cemented onto zirconia abutments are a good option for implant-supported single restorations in the anterior sector. The weakest part of the implant-abutment-crown complex in all groups was the fixing screw.
